# Neonatal Mitral Valve Repair in Biventricular Repair, Single Ventricle Palliation, and Secondary Left Ventricular Recruitment: Indications, Techniques, and Mid-Term Outcomes

**DOI:** 10.3389/fsurg.2015.00059

**Published:** 2015-11-10

**Authors:** Patrick O. Myers, Christopher W. Baird, Pedro J. del Nido, Frank A. Pigula, Nora Lang, Gerald R. Marx, Sitaram M. Emani

**Affiliations:** ^1^Cardiac Surgery, Boston Children’s Hospital, Harvard Medical School, Boston, MA, USA; ^2^Cardiovascular Surgery, Geneva University Hospitals and School of Medicine, Geneva, Switzerland; ^3^Cardiology, Boston Children’s Hospital, Harvard Medical School, Boston, MA, USA

**Keywords:** mitral valve, mitral valve repair, neonatal, congenital heart disease, surgery

## Abstract

**Objectives:**

Although mitral valve repair is rarely required in neonates, this population is considered to be at high risk for adverse outcomes. The aim of this study was to review the indications for surgery, mechanisms, repair techniques, and mid-term outcomes of neonatal mitral valve repair.

**Methods:**

The demographic, procedural, and outcome data were obtained for all neonates who underwent mitral valve repair from 2005 to 2012. The primary endpoints included mortality, transplantation, and mitral valve reoperation.

**Results:**

Twenty patients were included during the study period. Median age at operation was 11 days (range: 3–25). Eleven patients (55%) presented with mitral stenosis, three had regurgitation (15%), and six had mixed mitral disease (30%). Nineteen of 20 patients had mild or less regurgitation on immediate postoperative imaging. During a median follow-up of 5 months (1 month–4.8 years), six patients died at a median of 33 months (7–41 months) from repair and one patient required orthotopic heart transplantation. Six patients required mitral valve reoperation, five for mitral valve re-repair, and one for mitral valve replacement. Freedom from death, transplantation, or mitral valve replacement was 84.2 ± 8.4% at 1 month, 71.3 ± 11% at 6 months, 64.1 ± 12% at 1 year, and 51.3 ± 15% at 2 years and was worse for patients presenting with mitral regurgitation compared to stenosis or mixed mitral valve disease.

**Conclusion:**

Although mitral valve repair can be performed with acceptable immediate postoperative result, this procedure carries a high burden of late death and mitral valve reoperations.

## Introduction

Repair of mitral valve disease in children has been steadily increasing (1), and the results following complex reconstruction are improving (1–8). Congenital mitral valve disease rarely requires intervention in the neonatal period. When associated with hypoplasia of other left heart structures as part of borderline hypoplastic left heart syndrome, it may be managed by single ventricle palliation. With improving results in the management of congenital mitral disease in children and consideration for biventricular repair in borderline hypoplastic left heart syndrome, either through primary left ventricular rehabilitation (9) or secondary left ventricular recruitment (10), more patients are being referred for neonatal mitral valve repair. Very little data on this patient group is available (2). Valve repair in the neonate is technically challenging, and replacement with a mechanical prosthesis is associated with significant mortality (11). The aim of this study is to review the indications for surgery, mechanisms, repair techniques, and mid-term outcomes of neonatal mitral valve repair.

## Materials and Methods

### Study Design

This study is a retrospective review of all neonates who underwent mitral valve repair at our institution between 2005 and 2012. The demographic, procedural, and outcome data were obtained for all patients aged <30 days who underwent mitral valve repair during the study period. The primary endpoints included mortality, transplantation, mitral valve reoperation, and ≥moderate regurgitation or stenosis at follow-up. Clinical or treatment variables were recorded to determine predictors of the outcome measures. All patients underwent follow-up to death or June 2012. The study was approved by the Boston Children’s Hospital Institutional Review Board, and individual patient consent was waived.

### Surgical Technique

Cardiopulmonary bypass with moderate systemic hypothermia was used in all patients. Myocardial protection consisted of antegrade magnesium–lidocaine blood cardioplegia. The surgical techniques used for mitral valve repair were chosen by the operating surgeon, based on the mechanisms of mitral valve disease. The techniques utilized for repair were recorded from review of operative records.

### Statistical Analysis

Statistical analyses were performed with SPSS software (version 21, SPSS Inc., Chicago, IL, USA). Data are presented as mean ± SD or median (range) where appropriate. Continuous variables were analyzed with the Student’s *t*-test, or the related samples with Wilcoxon signed-rank test when appropriate, and categorical variables using the χ^2^ test or Fisher’s exact test. Actuarial estimates were calculated using the Kaplan–Meier method, and differences between curves were assessed by the log-rank test. All statistical tests were two-tailed and *P* values <0.05 were taken as significant.

## Results

### Demographics

Twenty patients were included during the study period. Patient baseline characteristics are summarized in Table 1. The median age at operation was 11 days (range: 3–25). Seven patients (35%) had associated hypoplastic left heart syndrome, six had critical aortic stenosis (30%), three had Shone’s complex (15%), one had unbalanced atrioventricular canal, and three had other congenital heart defects. Eight patients had a fetal intervention, six had postnatal balloon aortic valvuloplasty, and two had prior operations to address other congenital heart defects: aortic arch patch augmentation under low-flow cerebral perfusion and common atrioventricular canal defect repair in one patient, and coarctation repair, ventricular septal defect repair, and tricuspid valvuloplasty in the second patient. Indications for mitral valve repair included mitral stenosis in 11 patients (55%), regurgitation in 3 (15%), and mixed mitral disease in 6 patients (30%). Five patients were managed with single ventricle physiology: four patients with HLHS, and one patient with double outlet right ventricle and hypoplastic left ventricle and mitral valve; mitral valve repair was considered in the setting of staged left ventricular recruitment (10).

**Table 1:** **Baseline patient characteristics**.

Characteristic	Value
Age at mitral valve repair	11 days (range: 3–25)
Associated congenital cardiac anomalies	
Borderline HLHS	7 (35%)
Critical aortic stenosis	6 (30%)
Shone’s complex	3 (15%)
Unbalanced CAVC	1 (5%)
Other	3 (15%)
Type of predominant functional mitral disease	
Mitral stenosis	11 (55%)
Mitral regurgitation	3 (15%)
Mixed mitral disease	6 (30%)
Prior interventions	
Fetal balloon aortic valvuloplasty	7 (35%)
Fetal balloon mitral valvuloplasty	1 (5%)
Neonatal balloon aortic valvuloplasty	6 (30%)
Neonatal surgical operation	2 (10%)

*All data are presented as mean (SD) or number (percentage) unless otherwise noted*.

### Mitral Valve Repair

Mitral valve repair consisted of leaflet procedures in 8 patients (40%), annulus remodeling in 5 patients (25%), and subvalvular repair in 14 patients (70%). Techniques of valve repair are detailed in Table 2. Techniques used both in mitral stenosis and regurgitation have previously been described in detail (1, 2). In this subset of patients, most were managed with subvalvular repair techniques associated with leaflet repairs. Thickened leaflets were managed by thinning of endocardial fibroelastosis and opening of fused commissures by commissurotomy. The mobility of retracted leaflets was improved by thinning fused chords, splitting and mobilizing papillary muscles, and resection of secondary chords. Patch augmentation of deficient leaflets was rarely used in this subset of patients.

**Table 2:** **Operative characteristics**.

Variable	Number (%)
**Concomitant procedures**	
Stage 1 palliation of HLHS	4 (20)
Coarctation repair or arch augmentation	4 (20)
Aortic valvuloplasty	2 (10)
Ross–Konno	4 (20)
ASD repair	3 (15)
ASD restriction or creation	8 (40)
LV endocardial fibroelastosis resection	6 (30)
Subaortic obstruction resection	2 (10)
VSD repair	1 (5)
DORV repair	1 (5)
Aorto-pulmonary window repair	1 (5)
Biventricular repair after failed stage 1	1 (5)
**Mitral valve surgical techniques**	
Leaflet level repair	8 (40)
Commissurotomy	3 (15)
Leaflet tear closure	1 (5)
Cleft closure	1 (5)
Leaflet augmentation	1 (5)
Leaflet thinning	2 (10)
Annulus repair	5 (25)
Reed commissuroplasties	3 (15)
Suture annuloplasty	2 (10)
Subvalvular repair	14 (70)
Papillary muscle mobilization	8 (40)
Fused papillary muscles splitting	6 (30)
Fused chords thinning	1 (5)
Resection of secondary cords	5 (25)
Resection of accessory mitral tissue	1 (5)
Reimplantation of ruptured papillary muscle	1 (5)

*Data are presented as median (range) and number (percentage)*.

*ASD, atrial septal defect; HLHS, hypoplastic left heart syndrome; LV, left ventricular*.

On postoperative echocardiography, all patients had mild or trivial mitral regurgitation, with the exception of one patient with moderate regurgitation and a mean transmitral gradient of 1.4 ± 2.3 mmHg. One patient presented a transmitral gradient ≥7 mmHg.

### Outcomes

There were no hospital deaths (within 30 days of surgery or prior to discharge). During a median follow-up of 5 months (range: 1 month–4.8 years), six patients died at a median of 33 months (range: 1 month–3.4 years) from repair and one patient required orthotopic heart transplantation. The causes of death were low cardiac output in three patients, diffuse intravascular coagulopathy and liver dysfunction due to a possible storage disease in one patient, fungal sepsis in one patient, and pulmonary vascular disease in one patient.

Six patients required mitral valve reoperation, five for mitral valve re-repair, and one for mitral valve replacement. Two patients underwent mitral valve re-repair at bidirectional Glenn in the setting of secondary LV recruitment, and one patient underwent mitral valve re-repair for moderate stenosis (mean gradient 7.5 mmHg) during reoperation for recurrent left ventricular outflow tract and aortic valve obstruction, after fetal and neonatal aortic balloon valvuloplasty and neonatal surgical aortic valvuloplasty. Two patients underwent isolated mitral valve reoperation, one for recurrent mitral regurgitation due to a perforation in the anterior mitral leaflet at the site of insertion of an autologous pericardial patch augmenting the leaflet and one for recurrent mixed mitral disease after repair for congenital mitral and aortic stenosis. This patient’s first repair had included resection of secondary chords, leaflet thinning, and commissuroplasty. At reoperation, the anterior leaflet and posterior leaflets were thinned, secondary chords were divided, and fused papillary muscles were divided and mobilized from the ventricular wall. The patient who required mitral valve replacement originally had a repair for ischemic mitral regurgitation due to papillary muscle rupture. As the papillary muscle re-ruptured within 3 months with recurrent severe mitral regurgitation, the valve was replaced using a modified stented bovine internal jugular vein (Melody) valve in the mitral position (12, 13). This patient, who previously had severe left ventricular dysfunction which did not improve after mitral valve replacement, was listed for transplantation and subsequently underwent orthotopic heart transplantation.

**Figure 1:**
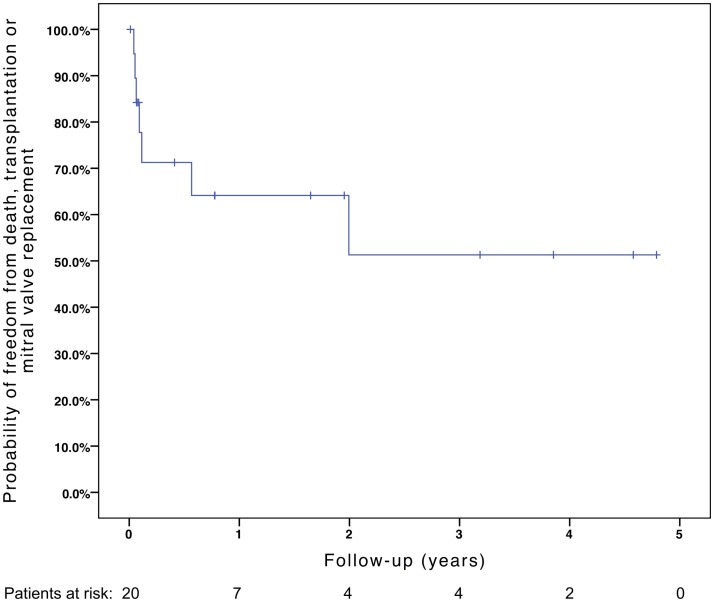
**Kaplan–Meier estimate of freedom from death, transplantation, or mitral valve replacement**.

**Figure 2:**
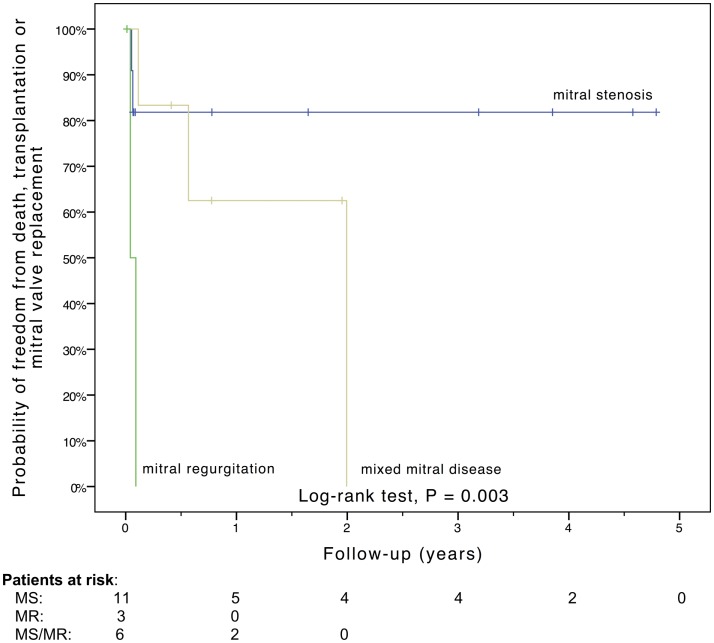
**Kaplan–Meier estimate of freedom from death, transplantation, or mitral valve replacement stratified by the mechanism of mitral valve dysfunction**. MR, mitral regurgitation; MS, mitral stenosis; MS/MR, mixed mitral disease.

**Figure 3:**
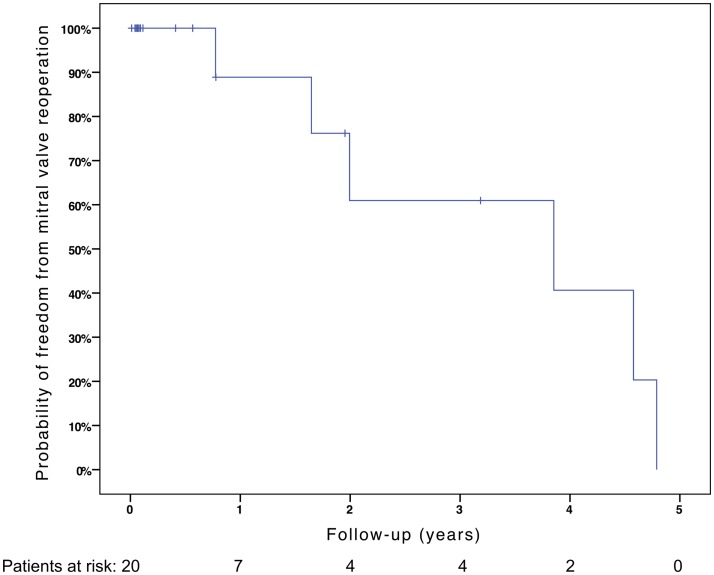
**Kaplan–Meier estimate of freedom from mitral valve reoperation**.

Freedom from death, transplantation, or mitral valve replacement was 84.2 ± 8.4% at 1 month, 71.3 ± 11% at 6 months, 64.1 ± 12% at 1 year, and 51.3 ± 15% at 2 years (see Figure 1). Freedom from this composite endpoint was 81.8 ± 11.6% at 1 month, 6 months, 1 year, and throughout follow-up in the 11 patients with mitral stenosis, 83.3 ± 15.2% at 1 and 6 months, 62.5 ± 21.3% at 1 year, and 0% at 2 years in the 6 patients with mixed mitral disease, and 50 ± 35.4% at 1 month and 0% at 6 months in the 3 patients with mitral regurgitation (*P* = 0.003, see Figure 2). When placing mixed lesions either in the group with stenosis or regurgitation based on the predominant mechanism, this difference between curves became non-significant (*P* = 0.72). Figure 3 provides the Kaplan–Meier estimates of freedom from mitral valve reoperation.

At the latest follow-up of transplant-free survivors (*n* = 13), seven patients demonstrate none to trivial mitral regurgitation, six patients demonstrate mild mitral regurgitation, and none have moderate or severe regurgitation. The mean transmitral gradient was 4.3 ± 2.7 mmHg (one patient with gradient >7 mmHg). With regards to pulmonary hypertension, one patient presented systemic pulmonary pressure after primary neonatal left ventricular rehabilitation for borderline HLHS (MS/AS), after neonatal mitral valve repair, subaortic stenosis resection, LV EFE resection, and aortic arch augmentation. This patient presented mild mitral regurgitation and no mitral stenosis on the latest echocardiogram. All other patients had no significant pulmonary hypertension.

Table 3 summarizes predictors of death, transplantation, or mitral valve replacement. Although there were minor differences in baseline and operative variables between patients who met the study endpoint versus those who did not, these differences were not statistically significant. Among patients who underwent stage 1 palliation of borderline HLHS, three did not reach the composite endpoint, while one died. Excluding these patients managed with single ventricle palliation, the median LV end-diastolic volume (LVEDV) and long axis *Z*-scores did not differ significantly between groups (LVEDV −1.69, range: −3.1 to 6.47 among patients not meeting the endpoint, versus 0.1, range: −1.81 to 2.14 among those reaching the endpoint, *P* = 0.37; LV long axis *Z*-score −1.16, range: −2.9 to 2.7 versus −0.42, range: −1.90 to −0.07, *P* = 0.84).

**Table 3:** **Predictors of death, transplantation, or mitral valve replacement**.

Risk factor	Endpoint-free	Endpoint	*P* value
Associated anomalies			0.17
HLHS	6 (46%)	1 (14%)	
Shone’s complex	3 (23%)	0 (0%)	
Unbalanced CAVC	0 (0%)	1 (14%)	
Critical aortic stenosis	3 (23%)	3 (43%)	
Other congenital cardiac anomaly	1 (8%)	2 (29%)	
Type of predominant functional mitral valve dysfunction			0.23
Stenosis	9 (69%)	2 (29%)	
Regurgitation	1 (8%)	2 (29%)	
Mixed mitral disease	3 (23%)	3 (43%)	
Prior interventions			
Fetal balloon aortic valvuloplasty	6 (46%)	1 (14%)	0.33
Fetal balloon mitral valvuloplasty	1 (8%)	0 (0%)	>0.99
Neonatal balloon aortic valvuloplasty	4 (31%)	2 (29%)	>0.99
Neonatal surgical operation	0 (0%)	2 (29%)	0.11
Left ventricular dimensions			
LVEDV Z-score	−2.1 (−4.71 to 6.47)	−1.36 (−4.25 to 2.14)	0.75
LV long axis dimension Z-score	−1.16 (−5.4 to 2.73)	−0.89 (−3.38 to −0.07)	0.82
Mitral valve repair surgical techniques			
Leaflet repair	6 (46%)	2 (29%)	0.64
Annulus remodeling	3 (23%)	2 (29%)	>0.99
Subvalvular repair	9 (69%)	2 (71%)	>0.99
Concomitant procedure			
Stage 1 palliation of HLHS	3 (23%)	1 (14%)	>0.99
Coarctation repair or arch augmentation	4 (31%)	0 (0%)	0.25
Aortic valvuloplasty	2 (15%)	0 (0%)	0.52
Ross–Konno	3 (23%)	1 (14%)	>0.99
ASD restriction	5 (39%)	2 (29%)	>0.99
LV EFE resection	5 (39%)	1 (14%)	0.35

*ASD, atrial septal defect; EFE, endocardial fibroelastosis; HLHS, hypoplastic left heart syndrome; LV, left ventricular*.

## Discussion

This retrospective review highlights a single institution experience with neonatal mitral valve repair. Subvalvular pathology is a predominant component of the mitral disease. Although postoperative outcomes are acceptable, reintervention is common. Children are increasingly being referred for valve repair, which likely results from the improving survival for complex congenital heart disease (5) and the focus on the development of valve repair and complex reconstruction rather than replacement (1). Valve repair in neonates is usually deferred as long as the patient can be managed medically, as repair is technically challenging, due to the greater fragility of the leaflet tissue, the often complex mechanism of mitral valve dysfunction in neonates, the significant mortality associated with valve replacement in small children (11), and the option to delay repair or choose single ventricle palliation, with proven results and outcomes (14).

There is an increased interest in neonatal mitral valve repair, with improving results in the management of congenital mitral valve disease in children (1, 2, 4–6), as well as the shortcomings of single ventricle palliation at long-term follow-up, and an increased interest in biventricular repair in borderline hypoplastic hearts (9, 10). Furthermore, delaying valve repair in selected patient groups is associated with worse outcomes: delaying valve repair in neonates with truncus arteriosus and truncal valve regurgitation is associated with an increased risk of mortality (15–17), while neonatal repair of this valve is associated with outcomes superior to infants or children undergoing truncal valve repair (15). Aortic valve repair in newborns has also been shown to have excellent results, which are superior to balloon valvuloplasty in neonatal aortic stenosis (18–20). These examples illustrate that aortic valve repair in the neonatal period is feasible and improves outcomes in selected difficult patient groups. The same needs to be assessed in other settings, such as congenital mitral disease.

As techniques for mitral valve in children have developed, so has the ability to apply these techniques to neonates as well as older children. Improvements in echocardiographic imaging allow the surgeon to assess the contribution of the various components of the mitral apparatus to overall valvular dysfunction. The high rate of immediate postoperative technical success demonstrated in this study is reflective of the improvements in imaging and techniques. However, in contrast to children and adult, the durability of the repair is suboptimal as demonstrated in this study. The reason for the poor durability is unclear, but may be related to accelerated degeneration of repair and high sensitivity to minor alterations in annular, leaflet, or papillary geometry.

Avoiding intervention in the neonatal period and deferring surgical management of the mitral valve until infancy may reduce the rate of reintervention, but raises the risk of progressive pulmonary vascular disease and interim mortality. In a study of 63 neonates with aortic coarctation and associated hypoplasia of either the aortic or mitral valve, who underwent biventricular neonatal repair with isolated coarctation repair, the mean mitral valve *Z*-score increased from −3.5 ± 1.9 at birth to +0.7 ± 1.6 at follow-up without intervention on the mitral valve (21). The strategy of single ventricle management may avoid the consequences of relying upon a diseased mitral valve for systemic output, but incurs the long-term risks of single ventricle physiology. An alternative strategy of single ventricle palliation to support the circulation while maneuvers are performed to repair the mitral valve allows conversion to biventricular circulation once the mitral disease has been stabilized (10). This strategy was applied for one patient in this series with anticipated biventricular conversion. Expanded use of this strategy may reduce the risk of pulmonary vascular disease and short-term mortality in patients with severe forms of mitral pathology. Improvements in mitral valve prostheses allow replacement to be a viable strategy for neonates (12, 13).

There is a paucity of data on neonatal mitral valve repair. Our group previously reported our experience in managing congenital mitral stenosis, with significantly worse freedom from reoperation in neonates than in infants and children (2). One report on neonatal mitral valve repair (22) described repair of *in utero* papillary muscle rupture in two neonates with tricuspid regurgitation and one 7-week-old infant with mitral regurgitation. Although there are numerous reports of mitral valve repair in children (1–4, 6, 23, 24), none of these have focused on or analyzed the subset of patients who required mitral valve repair in the neonatal period.

The present study reports the surgical repair of mitral valve disease in 20 neonates. These patients presented a mix of complex congenital heart disease, including Shone’s complex, borderline hypoplastic left heart syndrome, unbalanced common atrioventricular canal, critical aortic stenosis, or double outlet right ventricle, and five of these patients underwent single ventricle palliation. Mitral valve repair is composed of a mix of techniques, from simple commissurotomy to leaflet thinning, patch augmentation, and extensive subvalvular repair. Although there was a high burden of reoperation, using complex repairs, including on the leaflets or subvalvar apparatus, did not increase the rate of repair failure. Although a large proportion of our patients had congenital mitral stenosis, only one patient had patch augmentation with autologous pericardium. We previously described our institutional approach to this disease, which often involves incising the posterior leaflet from the annulus to gain access to the subvalvar apparatus, allowing mobilization of fused cords and papillary muscles (2). Patch augmentation of the posterior leaflet improves mobility, although mid- and long-term results of patch augmentation of the mitral valve is overall poor (25), due to limitations of currently available patch materials (26).

In this study, neonates with mitral regurgitation had worse outcomes compared to patients with mixed mitral disease or mitral stenosis. The data in older children would tend to show the inverse relationship (1). Our analysis may be confounded by the small sample size, and particularly the fact that just three patients had isolated mitral regurgitation. These patients tended to be sicker: two patients had severe biventricular dysfunction before mitral valve repair, and one patient required orthotopic heart transplantation and the other died secondary to liver dysfunction, with a suspected storage disease. The third patient had a relatively more simple disease (aorto-pulmonary window, mitral regurgitation due to a dilated mitral annulus and normal ventricular function), which was managed with suture annuloplasty at the time of aorto-pulmonary window repair, with a good outcome. The underlying severity of disease could be considered the reason for worse outcomes in this small group of patients with neonatal mitral regurgitation.

This study has several limitations. First, this was a retrospective, non-interventional study designed to evaluate the outcomes of an established clinical program. All patients were managed as individuals and not according to a treatment protocol, which would have improved our ability to analyze outcomes. Our analyses were limited by the limited patient sample, even if this represents one of the largest populations of neonatal mitral valve repair. Furthermore, the surgical techniques used included various techniques of mitral valve repair, as well as many differing underlying anatomies and associated anomalies or physiology, including single ventricle palliation, which may confound the analysis.

## Conclusion

Mitral valve repair can be done in neonates who require it, with low operative risk and allows valve preservation in a majority of patients at mid-term follow-up. This patient group carries a high burden of late death and mitral valve reoperations.

## Author Note

Presented at the STS 50th annual meeting in Orlando, FL on January 28th 2014.

## Conflict of Interest Statement

The authors declare that the research was conducted in the absence of any commercial or financial relationships that could be construed as a potential conflict of interest.
